# Tailoring Magnetite-Nanoparticle-Based Nanocarriers for Gene Delivery: Exploiting CRISPRa Potential in Reducing Conditions

**DOI:** 10.3390/nano13111782

**Published:** 2023-05-31

**Authors:** David Arango, Javier Cifuentes, Paola Ruiz Puentes, Tatiana Beltran, Amaury Bittar, Camila Ocasión, Carolina Muñoz-Camargo, Natasha I. Bloch, Luis H. Reyes, Juan C. Cruz

**Affiliations:** 1 Department of Biomedical Engineering, Universidad de Los Andes, Bogotá 111711, Colombia; d.arango@uniandes.edu.co (D.A.); jf.cifuentes10@uniandes.edu.co (J.C.); p.ruiz@uniandes.edu.co (P.R.P.); tc.beltran@uniandes.edu.co (T.B.); af.bittar@uniandes.edu.co (A.B.); c.munoz2016@uniandes.edu.co (C.M.-C.); n.blochm@uniandes.edu.co (N.I.B.); 2 Department of Chemical and Food Engineering, Universidad de Los Andes, Bogotá 111711, Colombia; c.ocasion10@uniandes.edu.co

**Keywords:** gene delivery, CRISPRa, magnetite nanoparticles, disulfide bond, *pink1*, nanoconjugates, cell delivery

## Abstract

Gene delivery has emerged as a promising alternative to conventional treatment approaches, allowing for the manipulation of gene expression through gene insertion, deletion, or alteration. However, the susceptibility of gene delivery components to degradation and challenges associated with cell penetration necessitate the use of delivery vehicles for effective functional gene delivery. Nanostructured vehicles, such as iron oxide nanoparticles (IONs) including magnetite nanoparticles (MNPs), have demonstrated significant potential for gene delivery applications due to their chemical versatility, biocompatibility, and strong magnetization. In this study, we developed an ION-based delivery vehicle capable of releasing linearized nucleic acids (tDNA) under reducing conditions in various cell cultures. As a proof of concept, we immobilized a CRISPR activation (CRISPRa) sequence to overexpress the *pink1* gene on MNPs functionalized with polyethylene glycol (PEG), 3-[(2-aminoethyl)dithio]propionic acid (AEDP), and a translocating protein (OmpA). The nucleic sequence (tDNA) was modified to include a terminal thiol group and was conjugated to AEDP’s terminal thiol via a disulfide exchange reaction. Leveraging the natural sensitivity of the disulfide bridge, the cargo was released under reducing conditions. Physicochemical characterizations, including thermogravimetric analysis (TGA) and Fourier-transform infrared (FTIR) spectroscopy, confirmed the correct synthesis and functionalization of the MNP-based delivery carriers. The developed nanocarriers exhibited remarkable biocompatibility, as demonstrated by the hemocompatibility, platelet aggregation, and cytocompatibility assays using primary human astrocytes, rodent astrocytes, and human fibroblast cells. Furthermore, the nanocarriers enabled efficient cargo penetration, uptake, and endosomal escape, with minimal nucleofection. A preliminary functionality test using RT-qPCR revealed that the vehicle facilitated the timely release of CRISPRa vectors, resulting in a remarkable 130-fold overexpression of *pink1*. We demonstrate the potential of the developed ION-based nanocarrier as a versatile and promising gene delivery vehicle with potential applications in gene therapy. The developed nanocarrier is capable of delivering any nucleic sequence (up to 8.2 kb) once it is thiolated using the methodology explained in this study. To our knowledge, this represents the first MNP-based nanocarrier capable of delivering nucleic sequences under specific reducing conditions while preserving functionality.

## 1. Introduction

Over the past decade, researchers have developed novel therapeutic agents to treat several diseases, particularly those lacking efficient treatment options. Even though these therapeutic agents have proved their safety in clinical trials, they often produce several side effects in patients [1,2,3]. These side effects, including nausea, gastrointestinal disturbances, and skin irritation, are attributed to the therapeutic agent or concomitant medications [4,5,6]. In the long term, side effects may necessitate additional efforts, such as medication changes or dosage increases, from patients and healthcare institutions [7,8]. Delivery methods have been used to mitigate some side effects related to the immune response, unspecific tissue delivery, and rapid molecule degradation [9,10]. However, certain therapeutic agents, such as poorly soluble molecules, large proteins, or genetic material, are less effective when administered through conventional delivery methods such as direct injections, systemic administration, or oral administration [11,12]. Consequently, there is a need for innovative delivery methods that address these limitations.

Gene delivery has emerged as a promising alternative treatment for many monogenic diseases, including Duchenne’s muscular dystrophy, sickle cell anemia, cystic fibrosis, hemophilia A, retinal dystrophy, and beta-thalassemia [13,14,15,16,17]. Despite the significant advances in recent years, gene therapy via gene delivery is still in its infancy, with several challenges to overcome before reaching the clinical stages. These challenges include avoiding DNA damage, the immune response, and component degradation [18,19]. These challenges can possibly be addressed by using novel gene carriers. Even though viral gene carriers have been previously used in gene delivery, they have shown undesirable effects in vivo, especially concerning patients’ safety and the limited size of the cargo [20,21]. Therefore, there is a need to develop novel gene carriers that can circumvent rapid degradation and the immune response while maintaining cargo functionality and a large cargo capacity.

Nanostructured vehicles have shown potential as gene delivery carriers due to their chemical versatility, stability, ease of preparation, and relatively low cost. Surface modification of nanocarriers, including alterations to their charge, chemistry, and hydrophobicity, can enhance stability and prevent rapid lysosomal degradation [22]. Additionally, nanostructured vehicles can be combined synergistically with other vehicles, adjuvants, or physical delivery strategies to maximize internalization and transfection efficiencies. One such strategy is PEGylation, which involves attaching polyethylene glycol (PEG) chains to nanocarriers, reducing clearance by the reticuloendothelial system (RES) and increasing the circulation time and bioavailability [23]. However, stringent international legislation and concerns about biocompatibility must be addressed before advancing to clinical applications [24,25]. Consequently, many research efforts are currently focused on elucidating the pharmacokinetics of delivery carriers, essential for successful gene delivery therapies [26,27].

Iron oxide nanoparticles (IONs), such as magnetite nanoparticles (MNPs), are promising for biomedical applications due to their homogeneous morphologies, surface charges, and sizes [24]. IONs have been employed in applications such as imaging, drug delivery, and tissue engineering [28]. One of IONs’ key features is their surface chemical versatility, allowing the conjugation of a wide range of molecules, including polymers, proteins, and peptides. This capability enables the delivery of therapeutic agents and imaging reagents, expanding the potential applications across various fields [29,30,31]. PEGylation is a popular functionalization strategy that enhances stability and prevents nanoparticle aggregation in physiological media [18]. These are all crucial attributes for in vitro and in vivo delivery applications [28]. Furthermore, ION functionalization with translocating proteins and peptides, such as the *Escherichia coli* outer membrane Protein A (OmpA) and the frog skin peptide Buforin II (BUF-II), can enhance cell internalization and cytosol coverage by evading endolysosomal trafficking pathways [30]. Another popular chemical modification involves pH-sensitive crosslinkers that facilitate cargo release under the cytosol’s typical reducing conditions [32].

In this work, we aimed to develop a nanostructured platform for immobilizing and intracellularly controlling the release of large (8.2 kb) linearized nucleic acids. The multifunctional nanostructured carrier, or nanobioconjugate, was designed based on the surface functionalization of MNPs with an organosilane molecule, followed by a polyethylene glycol (PEG) surface spacer, and a crosslinker molecule, (3-[(2-aminoethyl)dithio]propionic acid) AEDP, which contains a reducible disulfide linker in its structure. Simultaneously, a potent translocating and endosome-escaping protein called OmpA was co-immobilized to increase internalization and protect the biologically active genetic material from endosomal degradation. The reducible disulfide linker was exploited for the direct conjugation of a thiolated 65 bp tag to the free thiol on the surface of the conjugate, which is complementary to a non-encoding sequence within the delivery sequence (tDNA). This design enables the nanoparticle to potentially deliver any genetic material, as long as it contains a thiolated tag within its structure. This is made possible by the reducing conditions prevalent in the cytosol of cells, which facilitate the liberation of the cargo upon the entry of nanocarriers into the cellular environment.

As a proof of concept, we used the nanostructured vehicle to deliver linearized plasmid constructs of a CRISPRa system designed to overexpress the gene *pink1* in primary astrocytes, as a preliminary functionality test. We selected the gene *pink1*, which encodes for the PINK1 protein, due to its known function in the care and maintenance of mitochondria. Specifically, PINK1 regulates complex I function and prevents the accumulation of reactive oxygen species (ROS), which has been directly related to neurodegenerative diseases such as Parkinson’s disease [33]. By developing this nanostructured platform, we aim to advance the field of gene delivery and address some of the challenges associated with the current delivery methods for genetic material.

## 2. Materials and Methods

The authors conducted a thorough search for relevant information by accessing databases such as PubMed and ScienceDirect. They aimed to include research articles published from 2013 onwards, whenever possible. Additionally, prior articles authored by the researchers themselves and by the Biomedical and Chemical Engineering Departments of Universidad de los Andes were consulted and cited as references in this study.

### 2.1. Materials

Dimethyl sulfoxide (99.5%), tetramethylammonium hydroxide (TMAH) (25%), (3-aminopropyl) triethoxysilane (APTES) (98%), N-[3-(dimethylamino propyl]-N′-ethyl carbodiimide (EDC) (98%), glutaraldehyde (25%), NH2-PEG12-Propionic acid, Triton X-100, LB Broth Lennox, and Tween 20 were purchased from Sigma-Aldrich (St. Louis, MO, USA). Iron (III) chloride 6-hydrate pure, acetic acid (99.5%), and sodium hydroxide (NaOH) (98%) were obtained from Panreac AppliChem (Barcelona, Spain). Iron (II) chloride tetrahydrate (98%) was purchased from Alfa Aesar (Haverhill, MA, USA). Ampicillin CAS 69-53-4 was obtained from Santa Cruz Biotechnology (Dallas, TX, USA). NaCl was purchased from Loba Chemie (Mumbai, India). Pierce-AEDP (3-[(2-aminoethyl) dithiol] propionic acid) (AEDP) and TNBSA Solution (2,4,6-trinitrobenzene sulfonic acid) (5% *w*/*v*) were purchased from Thermo Fisher Scientific (Waltham, MA, USA). Dulbecco’s Modified Eagle Medium (DMEM) was purchased from Gibco (Amarillo, TX, USA), and Fetal Bovine Serum (FBS) was purchased from BioWest (Riverside, MO, USA).

Primers, sgRNA, the thiolated tag (used to bind the nanobioconjugate to the linearized plasmid), AgeI-HF Enzyme (NEB-R3552S), EcoRI-HF Enzyme (NEB-R3101S), PvuI-HF Enzyme (NEB-R3150S), Q5 High-Fidelity 2X Mastermix (NEB-M0515), Monarch PCR & DNA Cleanup Kit (NEB-T1030S), and Monarch Plasmid Miniprep kit (NEB-T1010S) were obtained from New England Bio labs (Ipswich, MA, USA). pCas-guide-CRISPRa (#GE100055) and pCas-Enhancer (#GE100056) were obtained from Origene (Rockville, MD, USA). Cell characterization was conducted in human primary dermal fibroblasts (HDFa, PCS-201-012) purchased from ATCC (St Cloud, MN, USA), and human astrocytes (NHA) were obtained from Lonza (Basel, Switzerland). A rat primary mixed culture (CP3A4, containing astrocytes, microglia, and neurons) was isolated, as described in the next section.

### 2.2. Isolation of CP3A4 Cells

Six neonatal Wistar rats (males of *Rattus norvergicus*, postnatal days 3–6) were bred in the Science Department at the Pontificia Universidad Javeriana, Bogotá, Colombia. The animals were housed in a colony room with a controlled temperature (24 ± 1 °C) and 12:12 h light/dark cycle, beginning at 10:00 A.M. The animal handling and sacrifice methods were reviewed and approved by the Committee for Animal Care and Use (FUA CICUAL Pontificia Universidad Javeriana F-CI-001-003-01).

Neonates were euthanized via decapitation, and their brains were promptly removed and placed in Hanks Balanced Salt Solution (HBSS) buffer containing 1% P/S. The cerebral cortices were carefully extracted and isolated from the rest of the brain. The specimens were inspected under a stereoscope to ensure complete whiteness, devoid of exposed veins or meninges. Following extraction, 300 mL of 10X trypsin was added to the samples and resuspended three times. The samples were then incubated for 20 min at 37 °C and 5% CO_2_ to enhance trypsin activity. Once heated, the samples were resuspended using pipettes with different volumes (10 mL, 5 mL, and 1 mL glass Pasteur pipettes) until fully homogenized. Immediately after the procedure, 2 mL of supplemented DMEM (10% FBS and 1% P/S) was added. The samples were centrifuged at 1200 rpm for 5 min to collect the cortex tissue, and the supernatant was carefully removed. This process was repeated as necessary. Next, 5 mL of DMEM was added to the solution, and the samples were resuspended until fully homogenized. Lastly, the sample was filtered through a 0.22-micron filter (Corning Falcon™). Cells were seeded at a density of 300,000 cells/mL and incubated under standard conditions. Additionally, cells were incubated on a stirring plate at 100 rpm under standard conditions for 24 h to complete the purification process. This process was repeated until the cells reached the desired purity.

### 2.3. MNP Synthesis

MNPs were synthesized via the co-precipitation of iron chlorides using the methodology described in a previous article [34]. A molar ratio of 2:1 between iron (III) chloride and iron (II) chloride was employed, and the chlorides were dissolved in type I water (water with a resistivity >1 MΩ-cm, and conductivity <1 µS/cm) to achieve concentrations of 200 mM and 100 mM, respectively. The chloride solution was prepared at 0 °C and added to a hermetically sealed round-bottom flask. Subsequently, 1 M NaOH solution was added dropwise to the chloride solution, which was then maintained under mechanical agitation at 200 rpm for 1 h and under constant nitrogen gas flow to sustain an inert atmosphere. Seven washing cycles involving precipitation (using 1.5% (*w*/*v*) NaCl) and resuspension were conducted to remove excess reagents. During this process, MNPs were magnetically precipitated using a powerful neodymium permanent magnet. Finally, a wash with Type I water was performed, suspending the MNPs in a salt-free medium.

### 2.4. Silanization and Surface Functionalization of MNPs

Silanization and surface functionalization of MNPs were performed, again using a previous article as reference [34], as follows: Briefly, 100 mg of MNPs was resuspended in type I water and sonicated for 5 min using a Branson 2800 Series Ultrasonic cleaner (Danbury, CT, USA). Subsequently, 2 mL of TMAH (25% *v*/*v*) and 50 µL of glacial acetic acid were added to the solution, which was then heated to 60 °C for 5 min. Next, 400 µL of APTES was added dropwise to the mixture and agitated at 220 rpm for 1 h. Nanoparticles were sonicated for 5 min between reagent additions. To remove impurities, nanoparticles were washed multiple times with NaCl solution and finally stored in type I water solution to prevent aggregation.

NH_2_-PEG_12_-Propionic acid (henceforth referred to as PEG) conjugation was carried out using glutaraldehyde as a crosslinker. Organosilane-MNPs (Sil-MNPs) were resuspended in 30 mL of type I water, and 2 mL of glutaraldehyde (2% *v*/*v*) was added to the solution. The reaction mixture was agitated at 180 rpm and maintained at room temperature for 30 min. PEG was then added at a 3:1 molar ratio relative to MNPs and allowed to react under agitation overnight. As previously described, the resulting PEG-MNP conjugates were washed with NaCl solution (pH 7) [34].

To form an amide bond between the PEG-free carboxyl groups and AEDP terminal amine groups, EDC was used as a crosslinker in the presence of NHS. The process involved dissolving 30 mg of EDC and 15 mg of NHS in an aqueous suspension of PEG-MNP, followed by heating the solution to 38 °C for 10 min to activate the carboxyl groups of PEG. Next, 5 mg of AEDP was added to the nanoconjugate solution, which was then agitated at 220 rpm overnight and washed with NaCl solution. The resulting AEDP-PEG-MNP nanoconjugates will be referred to as AEDP-MNP for simplicity.

For OmpA conjugation, 100 mg of the AEDP-MNP nanoconjugates was resuspended in 30 mL of type I water. A solution containing 30 mg of EDC and 15 mg of NHS dissolved in type I water was added to the mixture, which was then maintained at 38 °C for 10 min to activate the carboxyl groups of PEG. OmpA was subsequently added to the AEDP-MNP solution at a 3:1 molar ratio and agitated overnight. Finally, the obtained AEDP-MNP-OmpA nanobioconjugates were washed with NaCl and type I water, as described above.

### 2.5. Physicochemical Characterizations

The success of each conjugation step was verified using five different physicochemical characterization techniques. The hydrodynamic diameter was determined through dynamic light scattering (DLS) using a Zeta-Sizer Nano-ZS instrument (Malvern, UK) with 1 mL of a 1 mg/mL nanobioconjugate suspension in type I water. Moreover, the morphology and size in the solid state (after freeze-drying the samples) were analyzed via transmission electron microscopy (TEM) using a Tecnai F20 instrument with 200 kV as the operating voltage (FEI Company, Fremont, CA, USA). Surface charge (zeta potential) measurements were performed using the same Zeta-Sizer Nano-ZS instrument as mentioned earlier. Fourier-transform infrared (FTIR) spectroscopy was employed to confirm the presence of functional groups on the MNPs after each conjugation step. Measurements were taken using a Thermo Scientific Nicolet iS50 FTIR spectrometer (Waltham, MA, USA) with KBr pellets in the 400–4000 cm^−1^ wavenumber range. Thermogravimetric analysis (TGA) was conducted using a TA Instruments TGA Q50 (New Castle, DE, USA) to evaluate the weight loss and thermal stability of the nanoparticles throughout the synthesis process. Samples were heated from 30 °C to 800 °C at a rate of 10 °C/min under a nitrogen atmosphere.

### 2.6. Biological Testing

The biocompatibility of the AEDP-MNP-OmpA nanobioconjugates was evaluated through a series of biological tests, including hemolysis, platelet aggregation, and cytotoxicity assays, using a previously described methodology [34]. Erythrocytes (2 × 10^7^) were isolated from a healthy donor’s blood sample (obtained with the approval of the ethics committee of Universidad de los Andes, minute number 928-2018) via centrifugation, washed with 150 mM NaCl, and resuspended in 1 mL of 1X phosphate-buffered saline (PBS). A 96-well microplate was seeded in triplicate with 50 µL of the nanobioconjugates serially diluted from 400 µg/mL to 25 µg/mL, followed by the addition of 50 µL of the erythrocyte suspension to each well. A negative control containing 50 µL of 1X PBS and a positive control containing 50 µL of 1% *v*/*v* Triton X-100 were also included. Following a 1 h incubation at 37 °C, the microplate was centrifuged, and the absorbance was measured at 450 nm using a Multiskan FC Microplate reader (Thermo Fisher Scientific, Waltham, MA, USA). The hemolysis percentage (*H%*) was calculated according to Equation (1):

(1) $$H\%=\frac{OD_{test}-OD_{negative}}{OD_{positive}-OD_{negative}}*100$$

The platelet aggregation assay, based on the protocol of Cifuentes et al. [34], employed platelet-rich plasma (PRP) obtained from a healthy donor’s blood sample (collected under the permission granted by the ethics committee of Universidad de los Andes, minute number 928–2018) via centrifugation at 1000 rpm for 15 min. A 96-well microplate was seeded with 50 µL of the nanobioconjugate samples at concentrations ranging from 200 µg/mL to 12.5 µg/mL, followed by the addition of 50 µL of PRP to each well. The experiment was conducted in triplicate, with 1X PBS serving as a negative control and thrombin serving as a positive control. The absorbance was measured at 620 nm using a Multiskan FC Microplate reader, and the platelet aggregation percentage (*PA%*) was calculated using Equation (2):

(2) $$PA\%=\frac{OD_{test}-OD_{negative}}{OD_{positive}-OD_{negative}}*100$$

Cytotoxicity was assessed using a lactate dehydrogenase (LDH) assay on rat brain cortex astrocytes, human astrocytes (Lonza- CC-25659), and human primary dermal fibroblasts (ATCC-PCS-201-012). Cells were cultured in DMEM supplemented with 10% FBS and 1% P/S and incubated at 37 °C in 5% CO_2_. A total of 3 × 10^4^ cells were seeded in each well of a 96-well plate, and after 24 h of incubation, exposed to serial dilutions of the nanobioconjugates at concentrations ranging from 50 µg/mL to 3.125 µg/mL (DMEM without FBS) for 24 and 48 h. Following incubation, 50 µL of the supernatant and 50 µL of the LDH reaction mixture were transferred to a new 96-well microplate, and the LDH absorbance was measured at 490 nm and compared with the controls. Vero cells cultured in DMEM were used as a negative control, while 10% *v*/*v* Triton-X 100 served as a positive control. Experiments were conducted in triplicate.

### 2.7. Production of 3′Thiol-C6-SS-Modified DNA

**Scheme 1:**
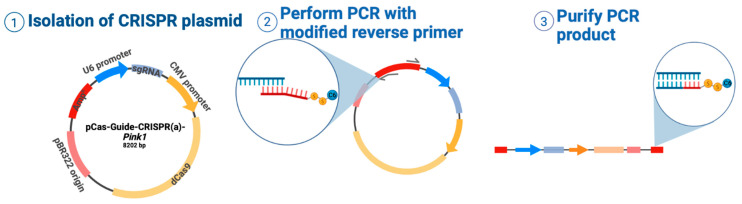
Schematic representation of tDNA production through PCR with the aid of a reverse modified (thiolated) primer.

**Figure A1:**
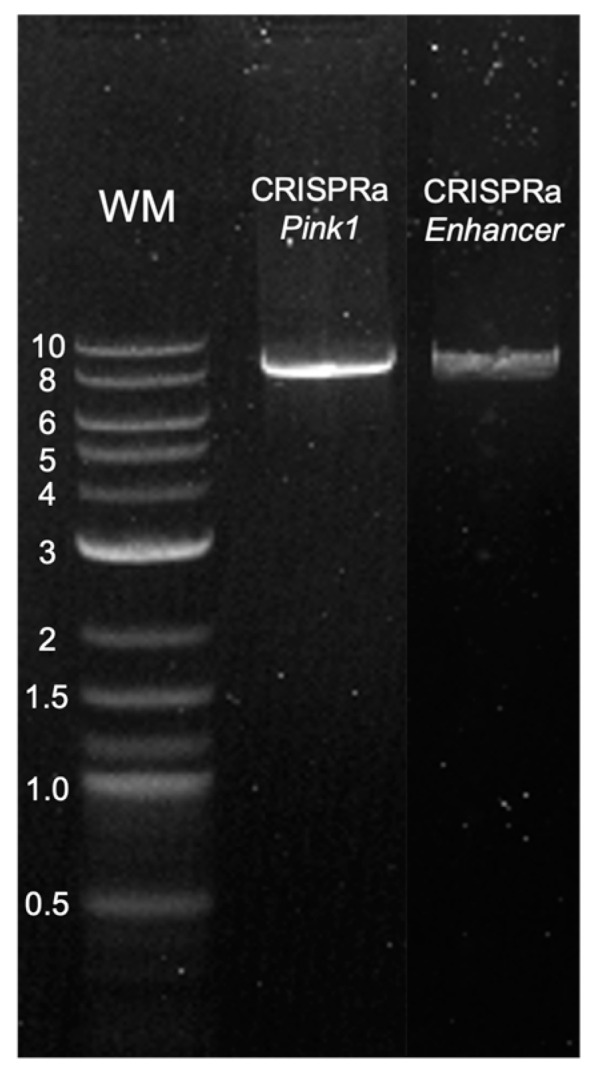
The electrophoresis gel confirmed a correct PCR product size of 7.8 kb. A molecular weight marker (WM) was run on the first lane. Lanes 2 and 3 are confirmations of the linear PCR products from the CRISPRa-*pink1* and CRISPR-*Enhancer* sequences, respectively.

To facilitate DNA conjugation with AEDP-PEG-MNP through disulfide bond formation, 3′ thiol-modified DNA (tDNA) was synthesized. The DNA release upon encountering the cytoplasm’s reducing conditions is mediated by the disulfide bond cleavage. PCR-based synthesis was performed using a CRISPR plasmid containing the sgRNA targeting the *pink1* gene, cloned in the pCas-guide-CRISPRa vector (referred to as the CRISPR plasmid; see Scheme 1) and transformed into DH5α *Escherichia coli* cells following an established protocol (described in Section 2.12). The CRISPR plasmid was extracted from an *E. coli* culture using a Monarch Plasmid MiniPrep kit (New England BioLabs, Ipswich, MA, USA). This plasmid contained an ampicillin resistance gene, sgRNA, and dCas9 sequences (Scheme 1). A 5′ Thiol-C6-SS-modified reverse primer with the sequence TCCTGCGTACGAGATGGAAATACTAGGTAACTACAGGGACTCCGcggaggac-cgaaggagctaac was used in the PCR. The last 21 bases of this reverse primer are complementary to the distal section of the ampicillin gene present in the CRISPR plasmid. The forward primer, which was unmodified, is complementary to the proximal sequence of the ampicillin gene in the CRISPR plasmid. PCR reactions were carried out using Q5 High-Fidelity MasterMix 2X (Eppendorf^®^ Mastercycler^®^ Nexus Thermal Cycler, Waltham, MA, USA) according to the manufacturer’s recommendations, with 30 ng of the DNA template, an annealing temperature of 67 °C, and an elongation time of 3 min and 20 s. The PCR product was purified using a Monarch PCR & DNA Cleanup Kit, and electrophoresis on 0.8% (*w*/*v*) agarose gel in 1X TBE confirmed the production of PCR tDNA. Ten PCR tDNA replicates were performed simultaneously to obtain sufficient genetic material for conjugation to the AEDP-PEG-MNP nanoconjugates. The results are shown in Figure A1.

### 2.8. Conjugation via Disulfide Bond Formation by Oxidation of Thiol Groups

**Scheme 2:**
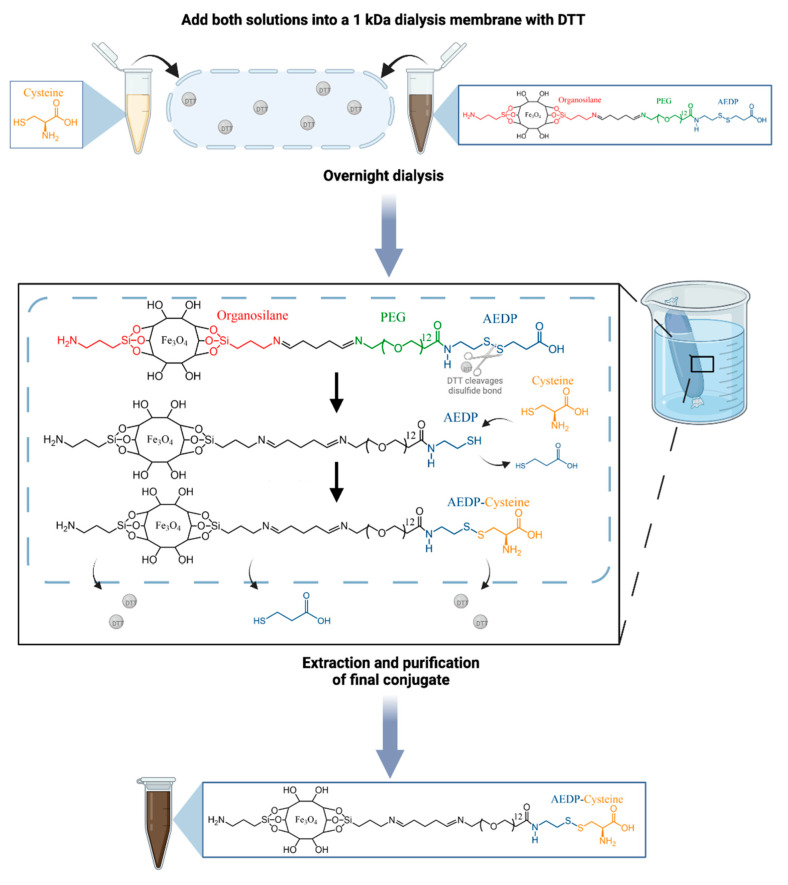
Schematic representation of the disulfide exchange reaction where cysteine is conjugated to the AEDP-MNP nanoconjugates by a disulfide bond.

The AEDP-MNP nanoconjugates contained a disulfide bond that was utilized for tDNA conjugation. The reduction of the disulfide bond generated terminal thiol groups on the conjugates, which, under oxidative conditions, reacted to form a disulfide bond with the tDNA. This reaction, known as disulfide exchange, has been widely used in various studies for thiol coupling [35]. As a proof of concept, disulfide exchange was first conducted using the thiol groups present in cysteine. Two conjugation reactions were completed: one with pure cysteine and another with Rhodamine B-labeled cysteine (Scheme 2). The latter allowed for tracking the delivery rate upon reduction by monitoring the corresponding fluorescent signal using a FluoroMax 4 spectrofluorometer (HORIBA Scientific, Piscataway, NJ, USA).

For the proof-of-concept cysteine conjugation, 250 µg of the AEDP-MNP nanobioconjugates was dissolved in 40 mM DTT solution, while a cysteine solution with a molar ratio of 7:1 (cysteine:AEDP-MNP) was prepared in 40 mM DTT. Then, 2.5 mL of each solution was added to a dialysis cassette equipped with a 1 kDa MWCO membrane for the thiol-coupling reaction. The dialysis was performed overnight against 100 mL of type I water, which was replaced every 2 h. As the process continued, the initial reducing conditions generated by DTT were gradually replaced by oxidizing conditions, resulting in the formation of Cys-AEDP-MNP nanoconjugates [36].

**Scheme 3:**
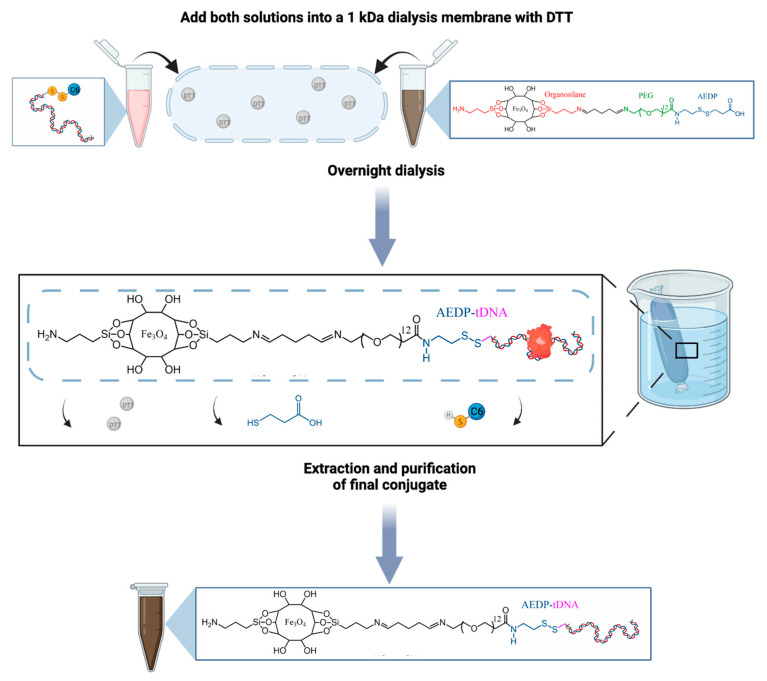
Schematic representation of the disulfide exchange reaction where the tDNA is conjugated to the AEDP-MNP nanoconjugates by a disulfide bond.

For the tDNA conjugation, 10 mg of the AEDP-MNP conjugates was dissolved in 5 mL of type I water, and 25 µg of tDNA was added to a separate 5 mL solution of 10 mM DTT. The same dialysis procedure as previously described was conducted, but with a 2 kDa MWCO dialysis membrane instead. After dialysis, the tDNA-AEDP-PEG-MNP nanoconjugates formed were transferred to a centrifuge tube and washed with NaCl solution, as described previously (Scheme 3). To prevent potential DNA degradation, the nanoconjugates were resuspended using brief sonication periods of 10 s at 37 kHz and 80 W in an ultrasonic bath (Elmasonic Easy 30 H, Elma Schmidbauer GmbH, Singen, Germany).

The OmpA translocating protein was then conjugated to the tDNA-AEDP-MNP nanoconjugates using the procedure mentioned above. This conjugation was performed at this stage to avoid possible detrimental changes in OmpA’s tertiary structure caused by the reducing conditions imposed by DTT.

### 2.9. In Vitro Delivery under Reducing Conditions

**Scheme 4:**
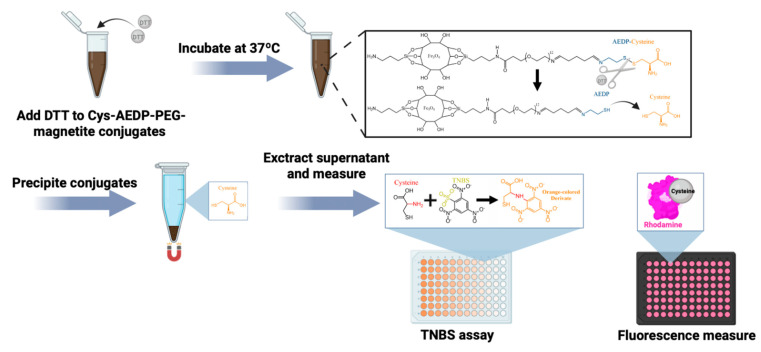
Schematic representation of the disulfide bond reduction and subsequent delivery procedure in the presence of the reducing agent DTT.

To assess the disulfide bond cleavage under reducing conditions, in vitro delivery of the Cys-AEDP-MNP nanoconjugates was conducted (Scheme 4). A mixture of 500 µL of the Cys-AEDP-MNP nanoconjugates (500 µg/mL) and 500 µL of DTT (20 mM) was incubated at 37.5 °C in an Eppendorf tube. The samples were collected at 0.5, 1, 2, 4, 6, 8, and 24 h of incubation to monitor the reaction kinetics. The Cys-AEDP-MNP nanoconjugates in the collected samples were magnetically precipitated, and 100 µL of the resulting supernatant was transferred to a 96-well microplate.

Disulfide bond reduction and subsequent cargo delivery were confirmed by two independent assays. The cysteine primary amine groups were quantified using trinitrobenzene sulfonic acid (TNBS). A glycine standard curve was prepared with serial dilutions ranging from 0.08 mg/mL to 0.005 mg/mL. Then, 100 µL of TNBS (0.05% (*v*/*v*)) was added to each microplate well, followed by a 2 h incubation at 37.5 °C. The absorbance was then measured at 405 nm using a Multiskan FC Microplate reader.

In an alternative approach, Rhodamine-labeled cysteine delivery was confirmed by fluorescence measurements using a FluoroMax Spectrofluorometer (Horiba, Piscataway, NJ, USA) with the excitation and emission wavelengths set at 548 nm and 578 nm, respectively, for the Rhodamine B fluorescence measurement. Since 100 µL of the supernatants was replaced by 100 µL of DTT, the data results were adjusted to account for dilution in the collected samples, using Equation (3):

(3) $$y_{t}=x_{t-1}+(0.7*x_{t})$$

### 2.10. Cell Delivery of tDNA-AEDP-MNP Nanoconjugates

To quantify the tDNA delivery in the cell cultures, tDNA was labeled with GelRed^®^ for spectrofluorometric measurements. A 10X GelRed^®^ solution was prepared in type I water, and 10 µL was added to 1000 ng of previously synthesized tDNA. The resulting mixture was incubated at 37 °C for 1 h.

The synthesized tDNA-AEDP-PEG-MNP nanoconjugates were delivered to NHA, HDFa, and CP3A4 cell cultures. Prior to conjugation, tDNA was fluorescently labeled with GelRed^®^ as described above. In 9.2 cm^2^ Petri dishes, 60,000 cells were seeded and supplemented with DMEM, 10% FBS, and 1% Penicillin/Streptomycin, followed by incubation at 37 °C in 5% CO_2_. Additionally, the nanoconjugates were co-delivered with the nuclear marker DAPI (1:1000) and endosomal marker LysoTracker Green^®^ DND-26 (1:10,000). Based on the observed disulfide bond cleavage kinetics, nanoconjugate delivery was conducted at 0.5, 4, and 7 h. Confocal imaging was performed using an Olympus FV1000 microscope with a UCPlan FL N 40×/0.6 objective. The excitation/emission wavelengths (nm) used for detecting nuclei, nanoparticles, and lysosomes were 405/461, 559/600, and 488/535, respectively. Fiji^®^ software was employed for image analysis. To prevent photobleaching, samples were consistently maintained and transported in containers and microplates wrapped in aluminum foil.

### 2.11. Functional Delivery Test

To evaluate the ability of the tDNA-AEDP-MNP-OmpA nanobioconjugates to release functional nucleic acids intracellularly, a functionality test of tDNA was conducted. A CRISPRa plasmid designed to activate *pink1* expression in CP3A4 cells was used. Two separate conjugates were constructed: one containing tDNA encoding for sgRNA (targeting *pink1*) and dCas9 (using pCas-guide-CRISPRa acquired), and another encoding for the CRISPRa-enhancer mechanism (using pCas-Enhancer acquired). The enhancer sequence produces the MS2, p65, and HSF1 proteins, which enhance gene activation by interacting with the VP64 transactivation domain. Relative expression changes induced by the delivered CRISPRa system were measured using real-time quantitative PCR (RT-qPCR).

### 2.12. sgRNA Design and CRISPRa System Construct

CRISPick^®^ software was employed to design sgRNAs targeting the *pink1* gene, with the lowest bioinformatic predictions for off-target activity being selected. An 8.2 kb origin plasmid, “CRISPRa—CRISPR/Cas9 SAM Synergistic Activation Mediator,” served as the base vector to engineer the final construct. Vector digestion was performed using the BamHI and BsmBI enzymes in a single reaction for 4 h at 37 °C, followed by a 16 h incubation in Cutsmart buffer at 55 °C. The vector was subsequently purified using the Monarch PCR & DNA Cleanup Kit. Ligation was carried out using the T4 ligase enzyme with a 16 h incubation at 16 °C. The final construct was transformed into *E. coli* DH5α, and the presence of sgRNA was validated via PCR. Finally, the sgRNA insert sequence was verified through Sanger sequencing.

### 2.13. RT-qPCR Quantification

CP3A4 cells were seeded at a density of 270,000 cells per well in a 12-well microplate and cultured in DMEM supplemented with 10% FBS. Cells were incubated at 37 °C in 5% CO_2_ for 24 h. Then, 50 µg of (CRISPRa-construct)-tDNA-AEDP-MNP-OmpA and 15 µg of (CRISPRa-enhancer)-tDNA-AEDP-MNP-OmpA were added to the cells in DMEM, while 65 µg of AEDP-MNP-OmpA was used as a control.

After incubation periods of 8, 24, and 48 h, cell RNA extraction was carried out using a Monarch Total RNA Miniprep Kit, following the manufacturer’s instructions with minor modifications. Cells were washed twice with PBS (1X), followed by the addition of 1 mL of TRIzol and thorough mixing via micropipetting. The mixture was transferred to an Eppendorf tube, and 200 µL of chloroform was added before centrifugation at 13,000 rpm for 15 min. The extraction protocol proceeded with 500 µL of the supernatant.

Subsequently, the Luna Universal One-Step RT-qPCR Kit protocol was followed, adding 33 µL of RNA to each reaction well in triplicate. GAPDH served as a reference gene, with the primer sequences being Forward-AGG GCT GCC TTC TCT TGT GAC AAA and Reverse-ATT CTC AGC CTT GAC TGT GCC GTT. The *pink1* primer sequences were Forward-ATC CAG CGG CAG TTC GTG GT and Reverse-TCC GCC TGC TTC TCC TCG ATC A. The Livak method was used for RT-qPCR data analysis [37].

## 3. Results and Discussion

### 3.1. Physicochemical Characterizations

**Figure 1:**
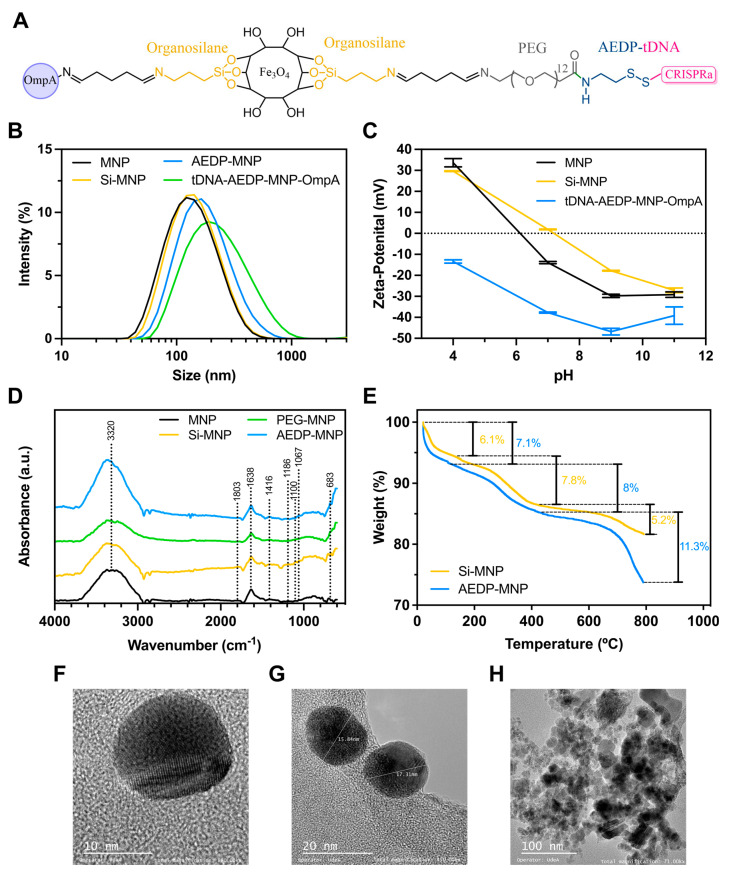
Physicochemical characterizations of magnetite, Si-MNP, and the final OmpA-AEDP-PEG-magnetite nanobioconjugates. (**A**) Chemical structure of the designed vehicle. (**B**) Hydrodynamic size distribution of MNPs, Si-MNP, AEDP-MNP nanoconjugate, and tDNA-AEDP-MNP-OmpA nanobioconjugate. (**C**) Zeta potential of the nanoconjugates. (**D**) FTIR spectra of MNP, Si-MNP, PEG-MNP, and AEDP-MNP nanobioconjugates. (**E**) TGA thermograms of the nanoconjugates and the corresponding weight loss percentages. (**F**,**G**) TEM images of individual magnetite nanoparticles. (**H**) TEM image of aggregates of the magnetite particles. Scale bars are 10 nm for figure (**F**), 20 nm for figure (**G**), and 100 nm for figure (**H**).

MNPs were successfully synthesized using the co-precipitation method [38,39,40]. The nanoconjugates’ average hydrodynamic diameters increased as functionalization progressed (Figure 1B). Size is a critical factor in nanocarrier delivery, as it can significantly influence the internalization mechanisms. Ideal diameters for delivery applications range between 20 nm and 1000 nm, with nanoparticles >20 nm effectively avoiding kidney clearance [41,42,43,44]. The average hydrodynamic diameter of the MNPs was 119 nm, with a polydispersity index (PI) of 19%, increasing to 132 nm and a PI of 20% for Si-MNP. The subsequent AEDP conjugation yielded AEDP-MNP nanoconjugates with an average size of 155 nm and a PI of 22%. The final tDNA-AEDP-MNP-OmpA nanobioconjugates exhibited an average hydrodynamic diameter of 199.2 nm and a PI of 34%, suitable for in vitro applications, as previously described [40,41]. The dynamic size distribution obtained is well-suited for gene therapy delivery applications [45]. However, 200 nm sized nanoparticles are predominantly internalized via endocytosis through clathrin- and caveolin-independent mechanisms, such as macropinocytosis and the Arf6-associated pathway [45]. This internalization often results in endosomal entrapment and subsequent loss of functionality. Incorporating translocating agents with endosome escape properties is crucial to address this issue. Our tDNA-AEDP-MNP-OmpA nanobioconjugates (Figure 1A) exhibit an increased likelihood of reaching the cytosol and delivering their cargo [30,46].

The observed decrease in the hydrodynamic diameter can be attributed to surface charges induced by PEG, OmpA, and tDNA [47]. The TEM micrographs (Figure 1F–H) revealed that the nanoconjugates possessed a typical spherical morphology [47,48], a critical parameter for efficient cellular uptake [49]. The micrographs showed that the average diameter of individual nanoparticles fell within the 15–17 nm range, consistent with previous reports for MNPs synthesized using the co-precipitation method [50,51,52]. DLS generally displays a larger particle size distribution than TEM because, in the former, the sample is suspended in an aqueous medium, while in the latter, it is dry [31].

Bare MNPs have been reported to exhibit a zeta potential distribution ranging from 30 mV at low pH values (i.e., 3 to 5) to −30 mV at high pH values (10 to 12), with an isoelectric point at around pH 6.4 (Figure 1C) [53,54]. This behavior can be attributed to the electrostatic interactions of the hydroxyl groups on the surface of MNPs with the H+ and OH− groups in the medium, depending on the pH [55]. ION isoelectric points have been reported to be near physiological pH (7), which suggests that nanoparticles aggregate and precipitate in such a medium due to the equilibrium of the electrostatic repulsion interactions [56]. The zeta potential distribution of our tDNA-AEDP-MNP-OmpA nanobioconjugates reached −37 mV at physiological pH, likely because of the negatively charged tDNA. This result aligns well with a previous report where, upon siRNA conjugation to gold nanoparticles, the Z-potential of the conjugates approached −50 mV [57,58]. Anionic nanoparticles typically fail to translocate to the cell membrane, probably due to repulsions with surface membrane proteins. As a result, their internalization is primarily governed by endocytosis mechanisms [57,59]. Owing to the anionic charge of the tDNA-AEDP-MNP-OmpA nanobioconjugates, we hypothesize that their direct cell membrane translocation is largely limited [46].

The effective immobilization and surface chemical structure of the delivery carrier were confirmed using Fourier-transform infrared (FTIR) spectroscopy (Figure 1D). The NPs exhibited a peak at 683 cm^−1^, attributable to the Fe-O vibration of magnetite [60]. This peak has been previously reported at 578 cm^−1^; however, a shift occurs due to the increased surface bond force resulting from the nanometric size of magnetite [61,62]. The FTIR spectrum of the Si-MNP conjugates displayed a peak at 1067 cm^−1^, assigned to the Si–O stretching vibration; at 1416 cm^−1^ due to the C-H bending vibration; and at 1100 cm^−1^, arising from the C-O bond from the conjugation of organosilane to the MNP core. Similarly, the PEG-MNP FTIR spectrum exhibited stretching at 3320 cm^−1^, associated with =N-H bonds resulting from the conjugation of organosilane and PEG. Additionally, the stretching vibration at 1186 cm^−1^ corresponds to the ether group C-O-C, present in PEG [63]. The AEDP-MNP spectrum showed an additional peak at 1803 cm^−1^, which can be associated with a stretching vibration of the C=O bond in the free carboxyl groups and amide bonds. A correct conjugation of AEDP by a disulfide bond must show stretching vibrations at 570 and 1725 cm^−1^ [64]. Unfortunately, the presence of hydroxyl groups and amide bonds (whose vibration is at 1640 cm^−1^) overlapped the 1725 cm^−1^ stretching, while a vibration of 570 cm^−1^ was not observed due to instrument limitations [65].

AEDP-MNP conjugation was also confirmed via thermogravimetric analysis (Figure 1E). The obtained thermograms exhibited an initial weight loss (20 °C to 150 °C) of 6.1% and 7.1% for the Si-MNP and AEDP-MNP conjugates, respectively. This weight loss can be attributed to sample dehydration. Moreover, the weight loss at 400 °C was 5.2% for Si-MNP and 11.3% for the AEDP-MNP nanoconjugate. This weight loss is ascribed to the release of immobilized molecules (Si, PEG, and AEDP). Additionally, the difference in weight loss (6.1%) between the silanization and conjugation with PEG and AEDP aligns well with previous work [22]. For MNPs, several reports indicate that their crystalline structure transitions to maghemite at 400 °C, altering their surface composition through the release of hydroxyl ions [65,66]. In the case of MNPs with a hydrophilic coating, at 400 °C, amine and organic bonds decompose [66,67,68,69,70,71].

### 3.2. Biological Testing

**Figure 2:**
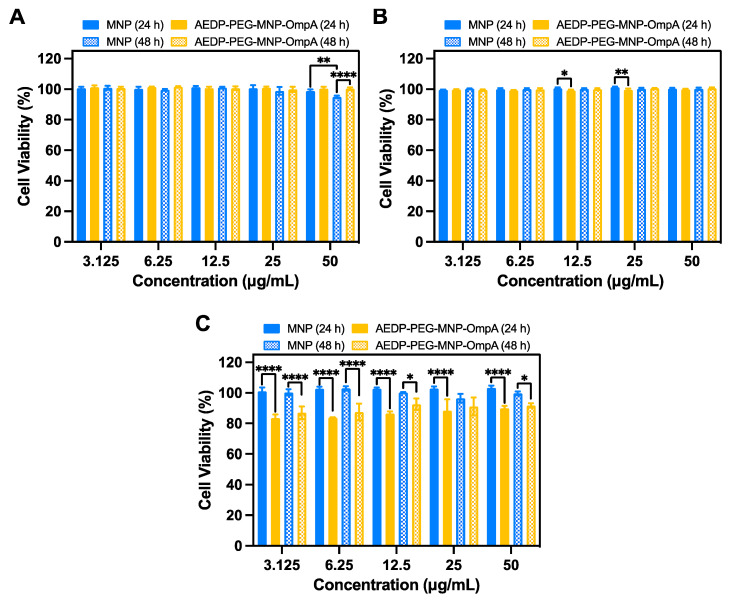
Cytocompatibility testing of the MNPs (black) and AEDP-MNP-OmpA (blue) nanoconjugates. Cell viability was evaluated at 24 (filled) and 48 h (pattern) in human astrocyte (NHA) (**A**), human dermal fibroblast (HDFa) (**B**), and rat primary mixed culture (CP3A4, containing astrocytes, microglia, and neurons) (**C**) cell cultures. DMEM was used as a negative control, and Triton X-100 (10% *v*/*v*) was used as a positive control. ANOVA and Tukey’s test were conducted for statistical analysis, using 5% as a significance level. * represents a significant difference with a *p*-value: 0.01 ≤ *p*-value ≤ 0.05; ** represents a significant difference with a *p*-value: 0.001 ≤ *p*-value < 0.01; **** represents a significant difference with a *p*-value < 0.0001.

The viability of two primary human cell lines, namely NHA and HDFa, and rat CP3A4 primary astrocytes (Figure 2A–C, respectively) was determined 24 and 48 h post-exposure to the MNPs and AEDP-MNP-OmpA nanobioconjugates via an LDH assay (Figure 2). The results showed cell viability above 95% after 48 h for both primary human cell lines (i.e., NHA and HDFa). Similar results were obtained for the rat primary astrocytes (CP3A4), with the viability exceeding 80% after 48 h of exposure to the MNPs and AEDP-MNP-OmpA nanobioconjugates at concentrations up to 50 µg/mL. These findings suggest high cytocompatibility, a prerequisite for in vivo testing [40,72].

**Figure 3:**
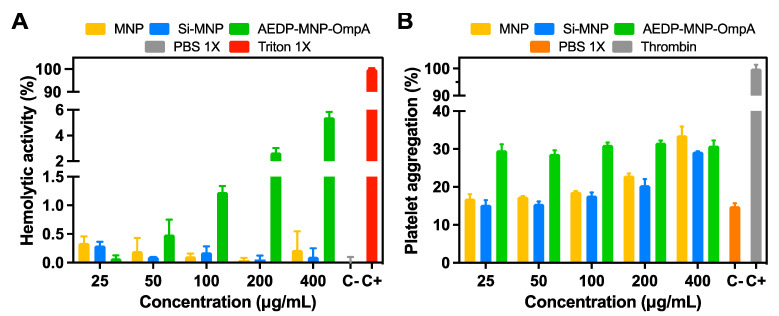
Hemocompatibility testing of the MNPs, Si-MNP, and AEDP-MNP-OmpA conjugates. (**A**) Hemolytic activity of the MNPs (black), Si-MNP (yellow), and AEDP-MNP-OmpA nanobioconjugates (blue) using PBS 1X as a negative control and Triton X-100 as a positive control. (**B**) Platelet aggregation of the MNPs, Si-MNP, and AEDP-MNP-OmpA nanoconjugates compared to PBS 1X (negative control) and thrombin (positive control).

The hemocompatibility of the nanobioconjugates was assessed through hemolysis and platelet aggregation assays (Figure 3A). The hemolytic activity of the MNPs and AEDP-MNP-OmpA nanobioconjugates was below 4% for concentrations up to 200 µg/mL, indicating a non-hemolytic material, in accordance with the ISO 10993-4 standard [60]. These results are comparable with those in the recent literature for MNPs and MNP-based conjugates [72].

A platelet aggregation test was also performed to determine the potential thrombogenic activity of the developed nanobioconjugates (Figure 3B). The platelet aggregation of the MNPs and Si-MNP nanoconjugates showed an aggregation of around 20% for concentrations below 200 μg/mL. Meanwhile, the AEDP-MNP-OmpA nanobioconjugates demonstrated slight aggregation (approximately 30%) at all evaluated concentrations. However, these results are comparable with those in recent reports on MNP-based delivery carriers [31,40]. Collectively, these assays indicate that the developed nanocarrier is highly biocompatible.

### 3.3. In Vitro Thiol Reduction and Delivery Testing

**Figure 4:**
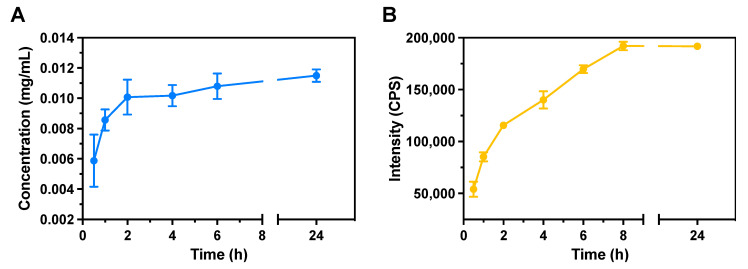
Disulfide reduction experiments for the delivery of both Rhodamine B-labeled cysteine and pure cysteine. (**A**) TNBS assay for quantifying the amine groups of the released cysteine. (**B**) Intensity of Rhodamine B fluorescence for the labeled cysteine delivered.

Cysteine was conjugated to AEDP-MNP using a thiol conjugation method to demonstrate immobilization and delivery under reducing conditions as a proof of concept. Figure 4A,B confirm the proper disulfide bond formation and subsequent cysteine release using DTT as a reducing agent in the medium, demonstrating successful disulfide bond formation between cysteine and tDNA through the proposed disulfide exchange reaction [46].

In vitro disulfide bond reduction was assessed through a TNBS assay and fluorescence measurements, revealing three distinct release rate regimes over the course of the experiment, each with decreasing delivery levels. The first regime occurred during the initial two hours, the second over the following four hours, and the third from that point onward until the experiment’s conclusion. The cumulative release amounted to approximately 86% of the initially loaded cysteine. Although both experiments exhibited similar trends, the minor differences may be due to the fluorescence measurements’ higher sensitivity compared to TNBS, which is more accurate for amino acid concentrations in the range of 20–200 µg/mL. For the intended application, delivery of the cargo within the first 8 h is likely sufficient to ensure an adequate circulation time and facilitate cell internalization [73].

Our proof-of-concept experiments indicate that our nanocarriers are promising options for tDNA delivery, as the disulfide bond is cleaved under reducing conditions [74]. Sensitive bonds for controlled release have frequently been incorporated into polymeric micelles and lipid-based nanoparticles; however, to our knowledge, our work is the first to feature MNPs modified with such moieties [75,76]. Studies have demonstrated that neurological disorders such as Parkinson’s disease cause a dramatic increase in reactive oxygen species (ROS) in neurons [77]. ROS can interact with disulfide bonds and potentially cleave them [78]. Furthermore, the low pH of endosomes and lysosomes may assist with digestion.

### 3.4. Cell Delivery

**Figure 5:**
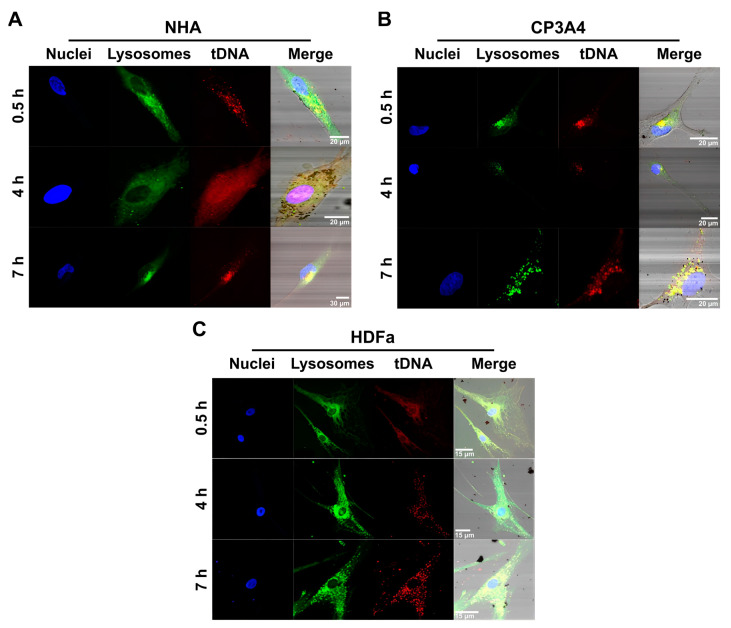
Confocal microscopy images of the internalization of the tDNA-AEDP-MNP-OmpA nanobioconjugates after 0.5, 4, and 7 h of incubation at 37 °C. Nuclei were labeled with DAPI dye, while endosome compartments were labeled with LysoTracker Green^®^. The experiment was performed in human astrocytes (NHA) (**A**), rat primary mixed culture cells (CP3A4, containing astrocytes, microglia, and neurons) (**B**), and human dermal fibroblasts (HDFa) (**C**). Scale bars are 10 µm for figures (**A**,**B**), and 15 µm for figure (**C**).

The developed nanovehicle was delivered into NHA, CP3A4, and HDFa cell cultures to evaluate its effectiveness in cell internalization (Figure 5A–C). Colocalization analysis between the labeled tDNA conjugated to the modified MNPs and endosomes tagged with Lysotracker Green^®^ enabled the calculation of the Pearson correlation coefficient (PCC), which ranges from 1 (perfect correlation) to −1 (perfect negative correlation). A similar analysis was performed for colocalization with labeled nuclei. Additionally, the nanovehicle’s distribution in the cytosol was assessed by calculating the percentage of area coverage.

**Figure 6:**
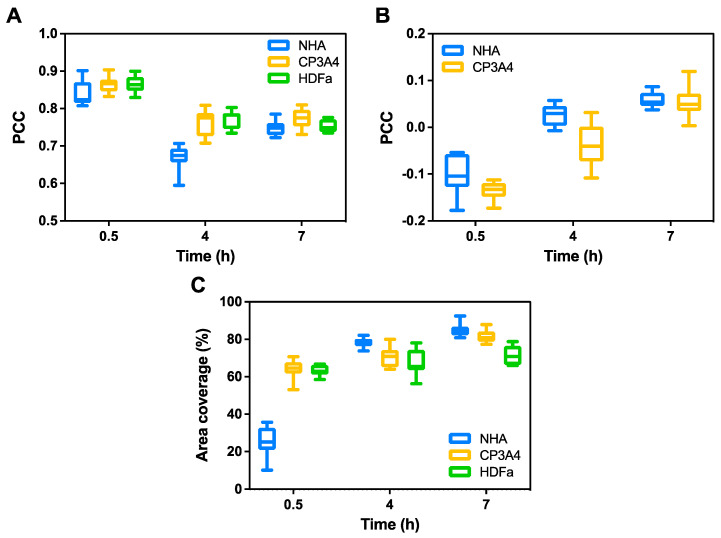
Cell delivery experiments of the tDNA-AEDP-MNP-OmpA nanobioconjugates. The endosome colocalization (**A**) and nuclei colocalization (**B**) results were estimated by calculating the Pearson correlation coefficient (PCC). Median significant differences are labeled with (*p* < 0.001) according to two-tailed t-tests using a 95% confidence level threshold. (**C**) The tDNA-AEDP-MNP-OmpA nanobioconjugate cell distribution according to the percentage of area covered by the tDNA.

Upon delivery, the cells exhibited no signs of nuclei damage, altered morphology, or apoptotic bodies, indicating that they remained healthy during the experiment, as previously evidenced in low-cytotoxicity observations. After 0.5 h, the PCC between tDNA and Lysotracker Green^®^ exceeded 0.85 for all evaluated cell cultures, signifying high endosomal entrapment (Figure 6A) [79]. The area covered by the nanovehicle after 0.5 h reached approximately 63% for the CP3A4 and HDFa cells (Figure 6C), while coverage for the NHA cells approached only 25%. After 4 h, the PCC decreased to around 0.75 and remained at that level after 7 h. This endosomal escape led to an increase in area coverage to about 80% after 7 h. Our previous work with MNP-OmpA and PEGylated-MNP nanobioconjugates demonstrated considerably lower PCC values in various cell cultures [40]. In this context, the size and surface charge of our tDNA-AEDP-MNP-OmpA nanobioconjugates may activate different internalization mechanisms. Due to their average hydrodynamic diameter of approximately 200 nm, the internalization of our nanobioconjugates might be primarily controlled by macropinocytosis and Arf6-associated endocytic pathways [80]. Moreover, a high negative surface charge (−37 mV) causes nanocarrier repulsion from membrane proteins, likely restricting direct cell membrane translocation, as previously reported [46].

The decrease in the PCC after 4 h suggests that a fraction of the MNPs escaped the endosomes. This relatively early endosome escape implies that the DNA cargo has a high likelihood of avoiding degradation and maintaining biological activity [81]. The “proton sponge effect” could be responsible for the reduction in endosome colocalization over time [46]. This effect is an endosome escape mechanism caused by the protonation of tertiary and, to a lesser extent, primary amines present in MNPs [81]. Despite the PCC remaining approximately constant after 4 h, the area increased by roughly 16%, 51%, and 4% for the CP3A4, NHA, and HDFa cells, respectively. This finding suggests that the tDNA-AEDP-MNP-OmpA nanobioconjugates may also be internalized through mechanisms distinct from endocytosis [82]. Future research will focus on investigating these mechanisms and their relative importance in detail.

Nuclear colocalization was also evaluated to obtain semi-quantitative information on the potential of tDNA nuclear transfection in both the CP3A4 and NHA cell cultures (Figure 6C). Due to variations in dye intensities and the scale of the involved molecular interactions, nuclear colocalization through confocal images must be corroborated quantitatively with molecular techniques (such as RT-qPCR and flow cytometry). Initially, the PCC between the nucleus and tDNA exhibited a slight anticorrelation (PCC < −0.1, at 0.5 h), indicating the absence of spatial colocalization. Over time, however, the PCC became less negative (PCC = [−0.067–0.02], after 4 h), finally becoming positive after 7 h (PCC = 0.058), which was significantly different from 0 (*p* < 0.001, t = 6.922, DF = 14, in a one-sample *t*-test with a 95% confidence level). This weak positive correlation indicates modest nuclear transfection of tDNA. Weak nuclear interactions are expected, given that OmpA-based nanovehicles have been observed in the nuclear region in previous studies [46,83]. Although nuclear transfection mechanisms require further investigation, these preliminary results are encouraging and suggest that tDNA is released into the cytosol and even reaches the nuclei. Quantitative analyses and flow cytometry should be conducted to unambiguously evaluate the transfection efficiencies.

### 3.5. Evaluation of pink1 Expression after CRISPRa Delivery

**Figure 7:**
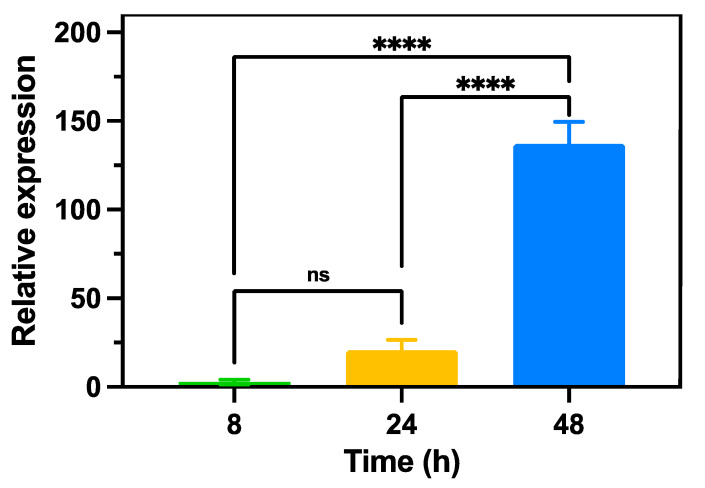
Time evolution of the relative expression of the *pink1* gene after delivery of the tDNA-AEDP-MNP-OmpA nanobioconjugates in rat primary mixed culture cells (CP3A4, containing astrocytes, microglia, and neurons). The *GAPDH* gene was used as a housekeeping gene for RT-qPCR analysis. ANOVA and Tukey’s test were conducted for statistical analysis, using 5% as a significance level. **** represents a significant difference with a *p*-value < 0.0001.

We assessed the capability of the tDNA-AEDP-MNP-OmpA nanobioconjugates to accurately deliver tDNA encoding CRISPRa elements into cells while maintaining biological activity to induce the anticipated overexpression of the *pink1* gene (Figure 7). We compared *pink1* expression in cells treated with CRISPRa elements specifically targeting *pink1*, conjugated to the AEDP-MNP-OmpA nanobioconjugates, against untreated control cells. Our findings indicated that, after 8 h, *pink1* expression was doubled in the treated cells compared to the control group. At 24 h, this difference increased to 25-fold, and after 48 h, *pink1* expression was approximately 130 times higher in the treated cells than in the control cells (Figure 7). These differences were statistically significant (*p* < 0.0001 after 48 h), providing robust evidence that the tDNA targeting *pink1* effectively transfects into cell nuclei, and the developed delivery vehicle is well-suited for gene delivery applications.

Our data demonstrate a continuous increase in *pink1* expression up to 48 h post-treatment, reaching the highest relative *pink1* expression, further corroborating the ability of the developed nanovehicle to prevent the rapid degradation of genetic material. Previous studies have reported gene expression changes induced through CRISPR-Cas systems, peaking after 24 h of exposure [84,85]. Another recent study employed viral vectors (AAV) to simultaneously target the transcription factors Asc1, Lmx1a, Nr4a2, and NeuroD1, involved in Parkinson’s disease, using CRISPRa for glial cell reprogramming. The expression of these transcription factors increased approximately 50-fold after 13 weeks [86]. Although future investigations on tDNA-AEDP-MNP-OmpA nanobioconjugates should focus on long-term effects, our study highlights the potential of our nanovehicles, as they offer the opportunity to efficiently deliver multiple nucleotide cargoes simultaneously.

## 4. Conclusions

We successfully synthesized and characterized MNP-based delivery carriers capable of performing gene delivery, penetrating cells, escaping endosomes, and delivering functional cargoes over a period of 48 h. This was achieved by conjugating nucleotide cargoes (tDNA) to MNPs functionalized with PEG, the disulfide-containing molecule AEDP, and the translocating protein OmpA. Our results demonstrate that the developed tDNA-AEDP-MNP-OmpA nanobioconjugates exhibit high biocompatibility, as evidenced by their cytocompatibility in two primary cell lines and hemocompatibility. As a proof of concept, the nanocarriers were employed to increase the expression of the *pink1* gene by 130-fold using a CRISPRa sequence, highlighting the nanocarriers’ ability to preserve the functionality of conjugated nucleotide sequences.

The developed nanovehicle is highly modular, allowing for the conjugation of any thiolated DNA sequence to the nanobioconjugates using the methodology described above. Furthermore, the responsiveness of the nanobioconjugates to the reducing conditions of the intracellular space makes them an attractive option for engineering multifunctional nanocarriers, as cargoes will only be delivered when the nanocarriers encounter reducing conditions. This innovative nanocarrier addresses the current limitations in drug and gene delivery, incorporating a unique combination of MNPs, translocating proteins, and reducing-sensitive cargo release. Further functionality tests should be conducted to confirm the preliminary results reported in this study. However, the modular nature of the developed carrier and its capability to deliver nucleic acids of varying sizes strongly indicate the immense potential of this nanocarrier in gene delivery applications.

Finally, we hypothesize that the overexpression of the *pink1* gene could be associated with the potential recovery of damaged mitochondria present in neurological diseases such as Parkinson’s. However, further studies should be conducted to target multiple genes simultaneously, to investigate gene expression changes and their relationship to Parkinson’s symptomatology. All in all, the impact of these MNP-based nanocarriers on gene delivery applications represents a significant advancement, offering promising potential for the targeted treatment of various diseases.

## Data Availability

Not applicable.
